# Increasing Vaccine Uptake during Pregnancy by Using Prenatal Education Classes: An Effective Tool for Health Communication and Promotion

**DOI:** 10.3390/children10091466

**Published:** 2023-08-28

**Authors:** Stefania Triunfo, Silvia Perossini, Esther Burdin, Elena Claudia De Angeli, Maria Francesi, Alessandra Garolfi, Jessica Moretti, Ilenia Paruscio, Miriam Tassielli, Marta Tremolada, Simona Gemelli, Deborah Pedrina, Anna Maria Marconi

**Affiliations:** Department of Obstetrics and Gynecology, ASST Santi Paolo e Carlo, S. Paolo University Hospital, Università degli Studi di Milano, 20122 Milan, Italy; silvia.perossini@gmail.com (S.P.); esther.burdin@asst-santipaolocarlo.it (E.B.); elenaclaudia.deangeli@asst-santipaolo.it (E.C.D.A.); maria.francesi@asst-santipaolocarlo.it (M.F.); alessandra.garolfi@asst-santipaolocarlo.it (A.G.); jessica.moretti@asst-santipaolocarlo.it (J.M.); ilenia.paruscio@asst-santipaolocarlo.it (I.P.); miriam.tassielli@asst-santipaolocarlo.it (M.T.); marta.tremolada@asst-santipaolocarlo.it (M.T.); simona.gemelli@asst-santipaolocarlo.it (S.G.); deborah.pedrina@asst-santipaolocarlo.it (D.P.); annamaria.marconi@asst-santipaolocarlo.it (A.M.M.)

**Keywords:** pregnancy, vaccine, health literacy, prenatal education classes

## Abstract

Childbirth education classes represent an antenatal tool for supporting pregnant women and couples in increasing knowledge on pregnancy, delivery, breastfeeding, and newborn care. The aim of this study was to investigate the impact of an additional lesson during the prenatal course regarding the advantage of vaccination to mitigation of maternal anxiety. An observational study was designed that included participants in childbirth education classes and compared courses enhanced by the extra lesson on vaccination during pregnancy versus those who did not receive it. Assessment of the impact of prenatal educational on vaccination was measured by using validated questionnaires (State-Trait Anxiety Inventory, STAI; Perceived Stress Scale, PSS; World Health Organization- Five Well-Being Index, WHO-5). A total of 145 pregnant women participated to the investigation by answering to the online survey. Of them, 33 patients (22.8%) belonged to the course without a lesson on vaccine, while 112 (77.2%) participated to online prenatal education that included an additional meeting on the usefulness of getting vaccinated during pregnancy. No statistical differences were found between study groups in terms of demographics and perinatal outcomes. Participants in the enriched course reported lower basal anxiety levels than those without the vaccine lesson (STAI-State, normal score < 40, 30 vs. 19%, *p*-value 0.041; STAI-State, mild score 40–50, 78 vs. 67%, *p*-value 0.037). With reference to the prior two weeks, maternal wellbeing level was improved by the added class (score > 13 as measurement of wellbeing: 62% vs. 80%, *p*-value < 0.05). Moderate perceived stress assessed by PSS was found in those pregnant women without prenatal education on vaccination (64 vs. 50%, *p*-value 0.042). The introduction of a lesson regarding vaccination during pregnancy in the program of prenatal education courses improved maternal anxiety levels and wellbeing, in addition to reducing perceived stress.

## 1. Introduction

Nowadays, vaccination is one of the most beneficial and cost-effective public health interventions, particularly when boosted vaccine uptake endorses immunization programs. In this scenario, a pivotal role might be played by vaccine hesitancy, defined as the reluctance or refusal to vaccinate despite the availability of vaccines, which is also listed as one of the ten threats to global health by the World Health Organization (WHO) [1].

In Italy, a significant and constant decrease in coverage from 2012 to 2017 for all vaccines at all age-groups has been reported. In response, the National Vaccination Prevention Plan 2017–2019 was planned as an effective and homogeneous strategy to be implemented throughout the country [2]. In this scenario, early programs to vaccinate against COVID-19 have recorded suboptimal coverage in specific geographical areas or among vulnerable population groups [3,4,5,6,7], although infection rates are high worldwide [8]. Multilevel strategies have been planned to enhance confidence in vaccination, reduce hesitancy, and expand vaccine uptake, mainly among people at higher risk. In Italy, an additional call to get vaccinated was promoted by the Conferenza Episcopale Italiana, the permanent assembly that assumes specific relevance in the relationships between the Italian State and the Catholic Church.

In obstetrics, the COVID-19 pandemic has emphasized the critical role of vaccination during pregnancy for minimizing the detrimental effects of infectious diseases on the feto-maternal dyad. However, pregnant women generally have a negative attitude about being vaccinated, as depicted in reports of flu and pertussis coverage during pregnancy [9,10]. Inadequate information, concerns about safety, fear of harming the fetus, and underestimation of the risks related to both illness and infection are the most common explanations for vaccine hesitancy. Bussink-Voorend stated that vaccine hesitancy represents “a psychological state of indecisiveness that people may experience when making a decision regarding vaccination”, regardless of their final choice [11]. A growing body of evidence has demonstrated the crucial role played by health professionals in this decision-making process as they are the most trusted sources of health- and vaccine-related information for pregnant women [12,13,14,15,16,17]. 

In Italy, in December 2021, the COVID-19 vaccine was initially only available to people in high-risk categories, such as health care professionals, nursing homes residents, highly vulnerable patients, and people aged ≥ 80 years [18]. At this stage, pregnancy was not considered a priority status and pregnant women were excluded from the vaccination call. At that time, no recommendation was made regarding vaccines to pregnant women from the Italian Obstetric Surveillance System (ItOSS). National recommendations only changed following medical literature that focused on the outcomes of SARS-CoV-2 infections worldwide, inclusive of the higher risk of stillbirth and/or other complications and severe illness among pregnant women with COVID-19, and that showed the safe and effective profile of the COVID-19 vaccine for both the mother and for the child [19,20,21,22,23,24]. Italian women of childbearing age started being vaccinated on 20 May 2021, followed by people aged 30–39 (on 27 May) and 12–29 (on 2 June); on 24 September, the Italian Ministry of Health published Circular No. 43293, indicating the safety of vaccination at any stage of pregnancy and during breastfeeding [25]. 

Unfortunately, the conflicting indications regarding COVID-19 vaccination during pregnancy has created fear and confusion among pregnant women over the time. This context emphasizes the importance of health care professionals’ advice and official recommendations for pregnant women [26,27,28]. A specific communication context requiring special communication skills has been emerging. Supplementary efforts have been arranged, varying from individual/collective counseling to dedicated open days for the vaccination of pregnant women, and also using novel tools for communication [29,30]. 

Childbirth education classes are antenatal health promotion and educational activities offered to pregnant women and their partners, with the aim of acting as public health and an empowerment tool [31]. In Italy, childbirth education classes can be offered both by public institutions and private providers. In absence of clear guidelines or standards, the program might include 3–7 sessions provided by a midwife, as well as other professionals (i.e., gynecologists, pediatricians, and anesthetists). The percentage of Italian women who attend childbirth education classes stands at 61.7% in the North, 55% in the Centre, 32.5% in the South, and 36.7% on the Islands, with significant reduction rates for foreign nationals (23.3%), mainly due to the limited number of courses offered in non-Italian languages [32,33]. 

On May 2021, the Italian Society of Obstetrics and Gynecology (SIGO) published a position paper with the aim of encouraging all pregnant women to get vaccinated against COVID-19, defining them as delicate people, and asking all health care professionals to get involved in prenatal care to promote vaccination by sharing updated evidence about the vaccine’s safety and efficacy [34]. 

Current prenatal education for parents regarding pregnancy, labor, birth, and neonatal care lacks ad hoc interventions on the usefulness to getting vaccinated during pregnancy against flu, pertussis, and COVID-19. Therefore, we aimed to explore the impact of an additional lesson on vaccination during pregnancy and to assess its impact on maternal anxiety by using well-established questionnaires.

## 2. Materials and Methods

### 2.1. Study Design, Population and Ethics

An observational study was designed that included participants in childbirth education classes, comparing courses enhanced by a supplementary lesson on vaccination during pregnancy with those that lacked it. 

San Paolo University Hospital in Milan offers a mixed prenatal course to all pregnant women that includes a total of 11 online and in-person lessons, that generally occur over 7 weeks and running from 30 to 36 weeks of gestation. The meetings had a median duration of 90 min, divided into a narrative and Q&A part, with each section lasting for 45 min. Different health professionals are involved, such as midwifes, obstetricians, pediatricians, anesthesiologists, are psychologists. The chosen topics include: 1. labor; 2. birth; 3. postnatal period; 4. early neonatal care; 5. at-home neonatal care; 6. preventive strategies for perineum damage; 7. pain relief strategies during labor; 8. medical interventions during labor; 9. focus on paternal role; 10. vaccination during pregnancy; and 11. emotional aspects during pregnancy, birth and postpartum. 

Since March 2022, a special lesson on vaccination during pregnancy was added to our prenatal education program. Overall, information was organized in slide presentations, were available for all participants at the end of the course, and included an email address for additional questions. Participants in the enriched course were allocated into the study group and were matched with those who were not exposed to the lesson or meeting on vaccination, who were defined as control group. The inclusion criteria were defined as meeting the following conditions: maternal age between 18–45 years, good comprehension of the Italian language, absence of psychiatric illness, availability of Internet access to participate in online meetings and receive electronic messages. The exclusion criteria were defined as meeting the following conditions: language barrier, maternal age less than 18 years, missed participation in the online lesson on vaccine, and poor completion of the questionnaires. 

All patients signed an informed consent form at the beginning of the course. The study was included in the project for assessing the performance of the quality of care (ASST Santi Paolo Carlo, n. 2170, September 2022).

### 2.2. Questionnaires and Measurements

By electronic message, an anonymous, well-defined questionnaire was sent to all participants. The questionnaire was designed in two sections:

- Part-A included items about maternal characteristics and perinatal outcomes (maternal age, ethnicity, country of birth, education level, working status, smoking status, medical and obstetric history, perinatal outcomes). Data reported by patients were confirmed by reviewing electronic medical records.
- Part-B assessed the impact of prenatal educational on vaccination, measured using validated questionnaires (State-Trait Anxiety Inventory, STAI; Perceived Stress Scale, PSS; World Health Organization- Five Well-Being Index, WHO-5) addressed to the participants. All tests administrated were Italian translation.

The STAI is a validated scale for scoring anxiety that is able to measure both trait anxiety (STAI-T) and state anxiety (STAI-S). An abnormal value of STAI was considered to be >40 [35]. 

The WHO-5 is one of the most sensitive and valid patient-reported outcome measures (PROMs) of subjective well-being, and it can be also used as a highly sensitive screening tool for depression. Time reference is related to the two weeks prior. The raw score ranges from 0 to 25, with 0 representing the worst possible and 25 representing the best possible quality of life [36].

The PSS is a stress assessment instrument, originally developed in 1983, and remains an option for helping researchers understand how different situations affect feelings and perceived stress during the last month. Individual scores on the PSS can range from 0 to 40, with higher scores indicating higher perceived stress. Scores ranging from 0–13 would be considered low stress; 14–26, moderate stress; and 27–40, high perceived stress [37].

### 2.3. Statistical Analysis

Descriptive statistics were calculated for the variables considered, and data are expressed as n (%) for categorical variables and median (standard deviation) for continuous variables. Chi-square or Fisher exact tests were used to compare group differences of categorical variables, and Wilcoxon or Mann–Whitney U tests were used for continuous variables. Pearson’s correlation was used to calculate the relationship between variables. A two-sided *p*-value of <0.05 was considered significant. 

## 3. Results

A total of 149 pregnant women were enrolled in the study; 4 (5.9%) of them were excluded after they declined to participate in the investigation, so the study population included 145 pregnant women willing to answer the online survey. Of them, 33 patients (22.8%) belonged to the prenatal education without the lesson on vaccine, while 112 (77.2%) participated in the prenatal education that included the additional class on usefulness of getting vaccinated during pregnancy.

In Table 1, maternal characteristics and pregnancy outcomes were reported. Both groups were similar, and no statistical differences were found between study groups in any demographic issues.

In Table 2, pregnancy outcomes were reported. No statistical differences were found between study groups in terms of perinatal outcomes. In particular, no additional complication at birth were found to be predominant in either group. 

**Figure 1:**
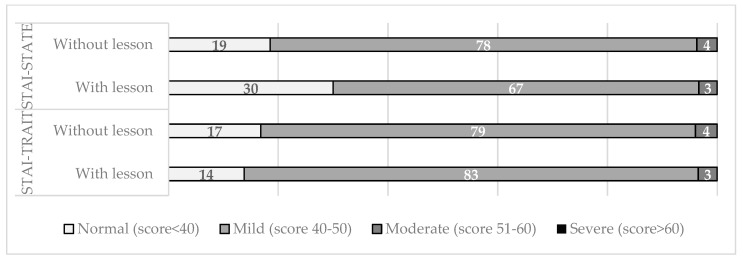
Distribution of maternal anxiety level, assessed by scores of STAI-Trait and STAI-State according to the presence or absence of the additional lesson on vaccination during pregnancy.

Participants in the enriched course reported lower anxiety levels than those without the lesson on vaccination during pregnancy. A significant decrease in basal conditions (STAI-S, normal score, 30 vs. 19%, *p*-value 0.041), and a decrease of mild levels (STAI-S, mild score, 78 vs. 67%, *p*-value 0.037) were calculated, as shown in Figure 1. 

**Figure 2:**
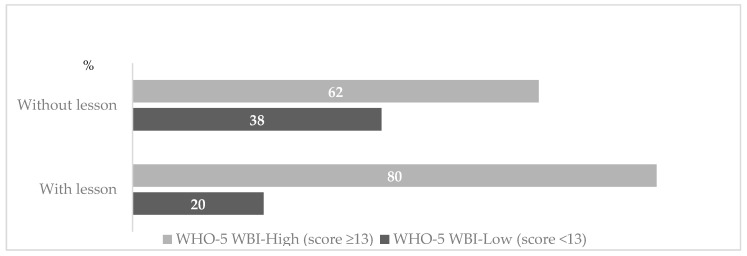
Distribution of maternal wellbeing level, assessed by WHO-5 WBI scores and according to the presence or absence of the additional lesson on vaccination during pregnancy.

During the last two weeks of the classes, maternal well-being level as assessed by WHO-5 WBI, as a short and generic global rating scale measuring subjective well-being, was improved by the added class on vaccination during pregnancy, as demonstrated by the raised scores (score > 13 as measurements of wellbeing: 62% vs. 80%, *p*-value < 0.05) (Figure 2).

**Figure 3:**
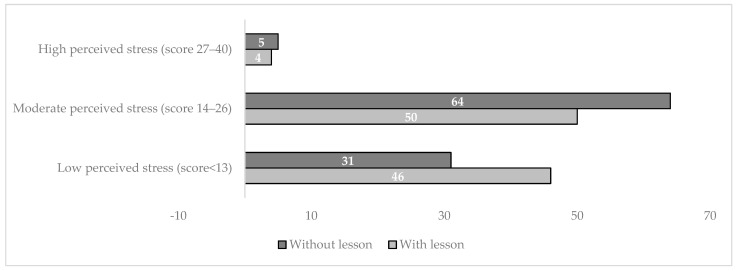
Distribution of maternal perceived stress levels, as assessed by scores of PSS and according to the presence or absence of additional lesson on vaccination during pregnancy.

Figure 3 shows moderate perceived stress, as assessed by PSS, in those pregnant women without prenatal education on vaccination (64 vs. 50%, *p*-value 0.042).

## 4. Discussion

To the best of our knowledge, this study shows for the first time the benefits on maternal anxiety, wellbeing, and perceived stress by prenatal education courses enriched by added lesson on getting vaccinated during pregnancy. As expected, among participants without information from health care providers, there was not only greater fear of abnormal perinatal outcomes induced by the vaccine, but also low scores of maternal wellbeing and moderate perceived stress over the time studied.

Infections in pregnancy can have deleterious effects on maternal and fetal health, largely dependent on the timing of infection. Among general and specific advantages of vaccination during pregnancy, the primary ones are the prevention of maternal morbidity and mortality and the reduction of risks of intrauterine infection and fetal disease [38]. The key mechanism that protects neonates from severe infective diseases is associated with the passive transfer of antibodies via the placenta and human milk [39].

Even though vaccination is one of the most cost-effective ways to avoid disease, it remains an open challenge. Estimates by the WHO report that immunization might prevent 4–5 million deaths every year in all age groups from diseases like diphtheria, tetanus, pertussis, influenza, and measles [40]. Additionally, if global vaccination coverage improves, an additional 1.5 million deaths could be avoided [40]. Vaccine hesitancy refers to the reluctance or refusal to vaccinate despite the availability of vaccines and represents one of the 10 threats to global health by the WHO [1]. There are several reasons why people choose not to vaccinate, such as complacency, lack of confidence, and inconvenience in accessing vaccines [41]. For that reason, it seems clear that health workers, especially in those communities, have the role of being the most trusted advisor and influencer of vaccination decisions in giving reliable information on vaccines [42]. In a previous study, we demonstrated an increase in vaccinations among pregnant women through monthly webinars based on the constant revisions of the medical literature, creating windows for asking and obtaining answers to personal apprehensions about safety, efficacy, and the rapid development and approval of the vaccines, as well as by holding events in partnership with one of the main vaccination centers in Milan, Italy [30].

Among pregnant women, an increased risk for some infective diseases has been described, mainly due the modifications characterized by moving from cell-mediated immunity, defined as Th1 response, to humoral immunity, defined as Th2 response [43]. As a coin with two sides, the adaptive change in maternal immunity includes two aspects: first, the protection of the semi-allogenic fetus from immunologic rejection; second, the increased susceptibility for pregnant women to severe conditions [44,45]. At the same time, pregnancy corresponds to the most delicate experience for women and couples, where the fear of damage the baby is endless, so much so any protective action believed to be harmful will be avoided regardless of its benefits.

Influenza infection in pregnancy is accompanied by increased maternal morbidity and adverse neonatal outcomes. The inactivated influenza vaccination administered during pregnancy can mitigate the potential detrimental effects of influenza in the newborn, as recommended by WHO [36], during the first months of life specifically [46]. Thus, before COVID-19, the influenza vaccine was the most widely recommended during pregnancy worldwide, mainly due the protective role played against potentially life-threatening disease in both pregnant people and their infants up to six months of age [47,48]. Despite this, only a few countries reported immunization rates in pregnant people above 50% [48]. 

Similarly, pertussis, an extremely contagious infection of the respiratory system that is caused by Bordetella pertussis, can affect individuals of all ages, with dangerous effects among neonates and children in particular. In Italy, reports from infant hospitalization for pertussis show that 64% of admissions are for those aged less than 1 year old [49]. To contrast these alarming rates and to promote the proper maternal production of antibodies, the National Vaccination Plan 2017–2021 by the Ministry of Health (and subsequent updates) recommend the administration of the trivalent vaccine (dTap) at the 28th gestational week ideally, or anytime between the 27th and 36th gestational weeks [2]. However, inadequate maternal understanding about vaccines, shaped by some unprecise indications from health professionals and amending legislative indications, and difficulty in receiving vaccine at the same time of consultations, has contributed to poor confidence in vaccination and the failure to achieve an adequate level of vaccination uptake among pregnant women. [50]

There is a growing body of evidence that the SARS-CoV-2 pandemic has increased maternal anxiety, and this condition may worsen the decision-making process on all vaccinations [6,7], including in those countries most affected by the first COVID-19 wave [30,51,52,53]. Our findings regarding the increase of the STAI-S scores on vaccination agrees with this overall trend. These findings might emphasize the impact of the vaccination process on mental health, mainly in women with a negative attitude toward the vaccine and unexposed to educational benefits by professionals in feto-maternal medicine. 

Childbirth education classes represent a health promotion and primary prevention tool within the antenatal support educational process. A growing body of evidence emphasizes the value of prenatal education classes routinely incorporated into maternity care for several reasons, such as maternal management of pain using a range of coping strategies [54], reducing cesarean section rates for the low-risk population [55], preparing for early parenting skills [56], perineal wound healing in postnatal women who experienced a tear, episiotomy, or both [57]. In Italy, antenatal education classes have been included in the national project on maternal and child health, called as ‘Progetto Obiettivo Materno Infantile—POMI’ [31], assigning a pivotal role to midwifes in the educational program in addition to their well-established support activities during delivery [58]. Our study adds knowledge on strategies for improving maternal wellbeing during pregnancy by suggesting revised methodological approaches of health literacy inclusive of additional tools. Due to being part of a fragile population, pregnant women remain a priority for vaccination coverage, and health care professionals are called upon to act against vaccine hesitancy by adding efforts to their current practices, in teamwork and by embracing social media, to reach the largest number of women of childbearing age.

The major strengths of the present study include a very well-characterized population of pregnant women who followed a structured intervention. Moreover, the use of three validated questionnaires with worldwide clinical applicability to assess anxiety, well-being, and perceived stress guaranties both rigor to the study and opportunity for comparison in all clinical settings. Additionally, the use of validated questionnaires may control not only the potential misclassification based on self-reporting data, but also the inherent risk of inaccuracy in the assessment. 

The study has some limitations. First, most women enrolled were Caucasians, belonging to a middle to high socio-economical level, without language barrier, and access to Internet; hence, our results could not be applied to other populations with different demographics. Secondly, online questionaries excluded partners from participation, even if they were involved during antenatal education. Third, the small sample size could require confirmation in more large study population.

## 5. Clinical Implications and Future Challenges

Based only on current actions, it is unlikely that this alone will offer significant success in overall vaccine up-take in pregnancy. Moving forward, strategies for addressing vaccine hesitancy among pregnant people require reevaluation. To design interventions based on multiple levels of the factors that influence vaccine hesitancy may offer more success than individual-level interventions alone. Prenatal education classes represent an effective educational solution to improve maternal awareness on the benefits of vaccination. Our findings encourage the inclusion of an extra lesson to the prenatal program for health care providers in all health service centers, irrespective of their public or private nature. Extra lessons can also be a promising tool for health literacy promotion on getting vaccinated during pregnancy.

## 6. Conclusions

The introduction of a lesson regarding vaccination during pregnancy in the program of prenatal education courses improves psychological well-being and mental health of pregnant women. Through a modest effort by health care professionals involved in prenatal education models, a great result in public health can be reached, both in terms of improved quality of life for pregnant women and in ameliorating immunization rates for newborns. As an intervention with a very low cost, health literacy on vaccination during pregnancy should be a fixed part of all prenatal education courses to improve the feto-maternal health from the beginning.

## Figures and Tables

**Table 1:** Maternal characteristics of the study population both with and without the additional lesson about vaccination during pregnancy in the childbirth education classes.

Variables	Absence of Lesson on Vaccines (*n* = 33)	Presence of Lesson on Vaccines (*n* = 112)	*p*-Value
Maternal age (years)	33.9 (4)	33.7 (5.1)	0.207
*Ethnicity*			
-Caucasian	32 (97)	106 (93.8)	0.007
-Latino-American	1 (3)	3 (2.7)	0.916
-Asian	0 (0)	2 (1.8)	0.443
-African	0 (0)	1 (0.9)	0.294
*Education Level*			
-Low -Medium -High	3 (9.1) 11 (33.3) 19 (57.6)	4 (3.6) 44 (39.3) 64 (57.1)	0.221 0.177 0.095
*Working status*			
-Employed -Unemployed	25 (75.8) 8 (24.2)	99 (88.4) 13 (11.6)	0.267 0.64
*Smoking status*			
-None -Smokers	29 (87.9) 4 (12.1)	97 (86.6) 15 (13.4)	0.956 0.121
Marital status			
-Married or living with partner -Unmarried	31 (93.9) 2 (6.1)	110 (98.2) 2 (1.8)	0.867 0.503
Alcohol abuse	0 (0)	0 (0)	-
Substance abuse	0 (0)	0 (0)	-

**Table 2:** Perinatal outcomes of the study population both with and without the additional lesson on vaccination during pregnancy in the childbirth education classes.

Variables	Absence of Lesson on Vaccines (*n* = 33)	Presence of Lesson on Vaccines (*n* = 112)	*p*-Value
*Medical condition*			
-Diabetes -Hypertension -Thyroid disease -Neurological disease -Autoimmune disease	2 (6.1) 1 (3) 2 (6.1) 2 (6.1) 1 (3)	5 (4.5) 3 (2.7) 15 (13.4) 4 (3.6) 4 (3.6)	0.721 0.916 0.297 0.547 0.916
*Parity*			
-Primiparous -Multiparous	22 (66.3) 11 (33.3)	87 (77.7) 25 (22.3)	0.622 0.329
Labor induction	11 (33.3)	46 (41.1)	0.592
*Mode of delivery*			
-Vaginal delivery -Instrumental delivery -Elective cesarean section -Emergent cesarean section	22 (66.6) 2 (6.1) 2 (6.1) 7 (21.2)	65 (58) 13 (11.6) 14 (12.5) 20 (17.9)	0.661 0.400 0.345 0.721

## Data Availability

Deidentified data will be shared upon request.

## References

[1] https://www.who.int/news/item/18-08-2015-vaccine-hesitancy-a-growing-challenge-for-immunization-programmes.

[2] https://www.salute.gov.it/imgs/C_17_pubblicazioni_2571_allegato.pdf.

[3] Scendoni R., Fedeli P., Cingolani M. (2023). The State of Play on COVID-19 Vaccination in Pregnant and Breastfeeding Women: Recommendations, Legal Protection, Ethical Issues and Controversies in Italy. Healthcare.

[4] De Brabandere L., Hendrickx G., Poels K., Daelemans W., Van Damme P., Maertens K. (2023). Influence of the COVID-19 pandemic and social media on the behaviour of pregnant and lactating women towards vaccination: A scoping review. BMJ Open.

[5] Gorgui J., Atallah A., Boucoiran I., Gomez Y.H., Bérard A., the CONCEPTION Study Group (2022). SARS-CoV-2 vaccine uptake and reasons for hesitancy among Canadian pregnant people: A prospective cohort study. CMAJ Open.

[6] Sarantaki A., Kalogeropoulou V.E., Taskou C., Nanou C., Lykeridou A. (2022). COVID-19 Vaccination and Related Determinants of Hesitancy among Pregnant Women: A Systematic Review and Meta-Analysis. Vaccines.

[7] Marín-Cos A., Marbán-Castro E., Nedic I., Ferrari M., Crespo-Mirasol E., Ventura L.F., Zamora B.N., Fumadó V., Menéndez C., Martínez Bueno C. (2022). “Maternal Vaccination Greatly Depends on Your Trust in the Healthcare System”: A Qualitative Study on the Acceptability of Maternal Vaccines among Pregnant Women and Healthcare Workers in Barcelona, Spain. Vaccines.

[8] https://www.worldometers.info/coronavirus/.

[9] Vilca L.M., Cesari E., Tura A.M., Di Stefano A., Vidiri A., Cavaliere A., Cetin I. (2020). Barriers and facilitators regarding influenza and pertussis maternal vaccination uptake: A multi-center survey of pregnant women in Italy. Eur. J. Obstet. Gynecol. Reprod. Biol..

[10] Maurici M., Dugo V., Zaratti L., Paulon L., Pellegrini M., Baiocco E., Rizzo G., Franco E. (2016). Knowledge and attitude of pregnant women toward flu vaccination: A cross-sectional survey. J. Matern. Fetal Neonatal Med..

[11] Bussink-Voorend D., Hautvast J.L., Vandeberg L., Visser O., Hulscher M.E. (2022). A systematic literature review to clarify the concept of vaccine hesitancy. Nat. Hum. Behav..

[12] Wilson R.J., Paterson P., Jarrett C., Larson H.J. (2015). Understanding factors influencing vaccination acceptance during pregnancy globally: A literature review. Vaccine.

[13] Agricola E., Gesualdo F., Alimenti L., Pandolfi E., Carloni E., D'Ambrosio A., Russo L., Campagna I., Ferretti B., Tozzi A.E. (2016). Knowledge attitude and practice toward pertussis vaccination during pregnancy among pregnant and postpartum Italian women. Hum. Vacc. Immunother..

[14] Myers K.L. (2016). Predictors of maternal vaccination in the United States: An integrative review of the literature. Vaccine.

[15] D’Alessandro A., Napolitano F., D’Ambrosio A., Angelillo I.F. (2018). Vaccination knowledge and acceptability among pregnant women in Italy. Hum. Vacc. Immunother..

[16] Poliquin V., Greyson D., Castillo E. (2019). A systematic review of barriers to vaccination during pregnancy in the Canadian context. JOGC.

[17] Scatigna M., Appetiti A., Pasanisi M., D’Eugenio S., Fabiani L., Giuliani A.R. (2022). Experience and attitudes on vaccinations recommended during pregnancy: Survey on an Italian sample of women and consultant gynecologists. Hum. Vacc. Immunother..

[18] https://www.epicentro.iss.it/vaccini/COVID-19-piano-vaccinazione.

[19] DeSisto C.L., Wallace B., Simeone R.M., Polen K.N.D., Ko J.Y., Meaney-Delman D., Ellington S. (2021). Risk for stillbirth among women with and without COVID-19 at delivery hospitalization—United States, March 2020–September 2021. MMWR Morb. Mortal. Wkly Rep..

[20] Conti M., Terreri S., Terrin G., Natale F., Pietrasanta C., Salvatori G., Brunelli R., Midulla F., Papaevangelou V., Carsetti R. (2022). SARS-CoV-2 infection versus vaccination in pregnancy: Implications for maternal and infant immunity. Clin. Infect. Dis..

[21] Tanne J.H. (2022). COVID-19: Vaccination during pregnancy is safe, finds large US study. Br. Med. J..

[22] De Rose D.U., Salvatori G., Dotta A., Auriti C. (2022). SARS-CoV-2 vaccines during pregnancy and breastfeeding: A systematic review of maternal and neonatal outcomes. Viruses.

[23] Dick A., Rosenbloom J.I., Gutman-Ido E., Lessans N., Cahen-Peretz A., Chill H.H. (2022). Safety of SARS-CoV-2 vaccination during pregnancy- obstetric outcomes from a large cohort study. BMC Pregnancy Childbirth.

[24] Ma Y., Deng J., Liu Q., Du M., Liu M., Liu J. (2022). Effectiveness and safety of COVID-19 vaccine among pregnant women in real-world studies: A systematic review and meta-analysis. Vaccines.

[25] https://www.iss.it/-/comunicato-stampa-n%C2%B068/2022-gravidanza-e-allattamento-vaccinazione-anti-COVID-19-aggiornate-le-indicazioni-dell-iss.

[26] Huddleston H.G., Jaswa E.G., Lindquist K.J., Kaing A., Morris J., Hariton E., Corley J., Hoskin E., Gaw S., Cedars M. (2022). COVID-19 vaccination patterns and attitudes among American pregnant individuals. Am. J. Obstet. Gynecol..

[27] Stuckelberger S., Favre G., Ceulemans M., Nordeng H., Gerbier E., Lambelet V., Stojanov M., Winterfeld U., Baud D., Panchaud A. (2021). SARS-CoV-2 vaccine willingness among pregnant and breastfeeding women during the first pandemic wave: A crosssectional study in Switzerland. Viruses.

[28] Tao L., Wang R., Han N., Liu J., Yuan C., Deng L., Han C., Sun F., Liu M., Liu J. (2021). Acceptance of a COVID-19 vaccine and associated factors among pregnant women in China: A multi-center cross-sectional study based on health belief model. Hum. Vacc. Immunother..

[29] Triunfo S., Marconi A.M. (2023). Promoting the Use of Modern Communication Tools to Increase Vaccine Uptake in Pregnancy. JAMA Pediatr..

[30] Triunfo S., Iannuzzi V., Podda M., Pedrina D., Gemelli S., Marconi A. (2023). Reducing vaccine hesitancy in pregnancy by the health literacy model inclusive of modern communication tools. Arch. Obstet. Gynecol.

[31] (2000). Progetto Obiettivo Materno Infantile.

[32] Grandolfo M.E., Donati S., Giusti A. (2002). Indagine conoscitiva sul percorso nascita. Aspetti Metodologici e Risultati Nazionali.

[33] ISTAT (2006). Gravidanza, Parto, Allattamento Al Seno 2004–2005.

[34] https://www.sigo.it/wpontent/uploads/2021/05/PositionPaper_Gravidanza_Vaccinazione_anti_COVID_05.05.2021.pdf.

[35] Spielberger C.D., Gorsuch R.L., Lushene R., Vagg P.R., Jacobs G.A. (1983). Manual for the State-Trait Anxiety Inventory.

[36] WHO (1998). Wellbeing Measures in Primary Health Care/The Depcare Project.

[37] Cohen S., Kamarck T., Mermelstein R. (1983). A global measure of perceived stress. J. Health Soc. Behav..

[38] Novillo B., Martínez-Varea A. (2022). COVID-19 Vaccines during Pregnancy and Breastfeeding: A Systematic Review. J. Pers. Med..

[39] Pillai A., Nayak A., Tiwari D., Pillai P.K., Pandita A., Sakharkar S., Balasubramanian H., Kabra N. (2023). COVID-19 Disease in Under-5 Children: Current Status and Strategies for Prevention including Vaccination. Vaccines.

[40] https://www.who.int/news-room/spotlight/ten-threats-to-global-health-in-2019.

[41] Demir R., Kaya Odabaş R. (2022). A systematic review to determine the anti-vaccination thoughts of pregnant women and the reasons for not getting vaccinated. J. Obstet. Gynaecol..

[42] Bisset K.A., Paterson P. (2018). Strategies for increasing uptake of vaccination in pregnancy in high-income countries: A systematic review. Vaccine.

[43] Formby B. (1995). Immunologic Response in Pregnancy: Its Role in Endocrine Disorders of Pregnancy and Influence on the Course of Maternal Autoimmune Diseases. Endocrinol. Metab. Clin. N. Am..

[44] Tan E.K., Tan E.L. (2013). Alterations in physiology and anatomy during pregnancy. Best Pract. Res. Clin. Obstet. Gynaecol..

[45] Mackin D.W., Walker S.P. (2021). The historical aspects of vaccination in pregnancy. Best Pract. Res. Clin. Obstet. Gynaecol..

[46] Iuliano A.D., Roguski K.M., Chang H.H., Muscatello D.J., Palekar R., Tempia S., Cohen C., Gran J.M., Schanzer D., Cowling B.J. (2018). Estimates of global seasonal influenza-associated respiratory mortality: A modelling study. Lancet.

[47] Lafond K.E., Porter R.M., Whaley M.J., Suizan Z., Ran Z., Aleem M.A., Thapa B., Sar B., Proschle V.S., Peng Z. (2021). Global burden of influenza-associated lower respiratory tract infections and hospitalizations among adults: A systematic review and meta-analysis. PLoS Med..

[48] Regan A.K., Fiddian-Green A. (2022). Protecting pregnant people & infants against influenza: A landscape review of influenza vaccine hesitancy during pregnancy and strategies for vaccine promotion. Hum. Vaccin. Immunother..

[49] Brosio F., Kuhdari P., Cocchio S., Stefanati A., Baldo V., Gabutti G. (2020). Impact of pertussis on the Italian population: Analysis of hospital discharge records in the period 2001–2004. Int. J. Infect. Dis..

[50] Gabutti G., Cetin I., Conversano M., Costantino C., Durando P., Giuffrida S. (2022). Experts’ Opinion for Improving Pertussis Vaccination Rates in Adolescents and Adults: A Call to Action. Int. J. Environ. Res. Public Health.

[51] Skjefte M., Ngirbabul M., Akeju O., Escudero D.J., Hernández-Díaz S., Wyszynski D., Wu J. (2021). COVID-19 vaccine acceptance among pregnant women and mothers of young children: Results of a survey in 16 countries. Eur. J. Epidemiol..

[52] Mappa I., Luviso M., Distefano F.A., Carbone L., Maruotti G.M., Rizzo G. (2022). Women perception of SARS-CoV-2 vaccination during pregnancy and subsequent maternal anxiety: A prospective observational study. J. Matern. Fetal Neonatal. Med..

[53] Engjom H., van den Akker T., Aabakke A., Ayras O., Bloemenkamp K., Donati S., Cereda D., Overtoom E., Knight M. (2022). Severe COVID-19 in pregnancy is almost exclusively limited to unvaccinated women—Time for policies to change. Lancet Reg. Health Eur..

[54] Escott D., Slade P., Spiby H. (2009). Preparation for pain management during childbirth: The psychological aspects of coping strategy development in antenatal education. Clin. Psychol. Rev..

[55] Ronco S. (2021). Literature Review of the Association Between Prenatal Education and Rates of Cesarean Birth Among Women at Low Risk. Nurs. Womens Health.

[56] Lau R., Hutchinson A. (2020). A narrative review of parental education in preparing expectant and new fathers for early parental skills. Midwifery.

[57] O’Kelly S.M., Moore Z.E. (2017). Antenatal maternal education for improving postnatal perineal healing for women who have birthed in a hospital setting. Cochrane Database Syst Rev..

[58] Ricchi A., La Corte S., Molinazzi M.T., Messina M.P., Banchelli F., Neri I. (2020). Study of childbirth education classes and evaluation of their effectiveness. Clin. Ter..

